# A randomised sham controlled trial of vertebroplasty for painful acute osteoporotic vertebral fractures (VERTOS IV)

**DOI:** 10.1186/1745-6215-12-93

**Published:** 2011-04-05

**Authors:** Cristina Firanescu, Paul NM Lohle, Jolanda de Vries, Caroline A Klazen, Job R Juttmann, William Clark, Willem Jan van Rooij

**Affiliations:** 1 Department of Radiology, St. Elisabeth Ziekenhuis, Hilvarenbeekseweg 60, 5022GC Tilburg, The Netherlands; 2 Department of Internal Medicine, St. Elisabeth Ziekenhuis, Hilvarenbeekseweg 60, 5022GC Tilburg, The Netherlands; 3 Department of Medical Psychology, St. Elisabeth Ziekenhuis, Hilvarenbeekseweg 60, 5022GC Tilburg, The Netherlands; 4 Department of Radiology, Medisch Spectrum Twente, Haaksbergerstraat 55, 7513 ER Enschede, The Netherlands; 5 Department of Radiology, St. George Private Hospital, 1 South Street, Kogarah NSW 2217, Australia; 6 Department of Medical Psychology, Tilburg University, Warandelaan 2, 5037 AB Tilburg, The Netherlands

## Abstract

**Background:**

The standard care in patients with a painful osteoporotic vertebral compression fracture (VCF) is conservative therapy. Percutaneous vertebroplasty (PV), a minimally invasive technique, is a new treatment option. Recent randomized controlled trials (RCT) provide conflicting results: two sham-controlled studies showed no benefit of PV while an unmasked but controlled RCT (VERTOS II) found effective pain relief at acceptable costs. The objective of this study is to compare pain relief after PV with a sham intervention in selected patients with an acute osteoporotic VCF using the same strict inclusion criteria as in VERTOS II. Secondary outcome measures are back pain related disability and quality of life.

**Methods:**

The VERTOS IV study is a prospective, multicenter RCT with pain relief as primary endpoint. Patients with a painful osteoporotic VCF with bone edema on MR imaging, local back pain for 6 weeks or less, osteopenia and aged 50 years or older, after obtaining informed consent, are included and randomized for PV or a sham intervention. In total 180 patients will be enrolled. Follow-up is at regular intervals during a 1-year period with a standard Visual Analogue Scale (VAS) score for pain and pain medication. Necessary additional therapies and complications are recorded.

**Discussion:**

The VERTOS IV study is a methodologically sound RCT designed to assess pain relief after PV compared to a sham intervention in patients with an acute osteoporotic VCF selected on strict inclusion criteria.

**Trial registration:**

This study is registered at ClinicalTrials.gov., NCT01200277.

## Background

Because of an aging population, osteoporosis and associated fractures are becoming an important health issue, especially in Western societies. The incidence of a new vertebral compression fracture (VCF) in Europe is, at age 50-79 years, 1% per year in women and 0.6% per year in men and at age 75-79 years the incidence is 2.9% per year in women and 1.4% per year in men [1]. VCFs are associated with an increased incidence of mortality and morbidity, including back pain, loss of height, kyphotic deformity and a reduction in quality of life [2].

Conservative therapy, bed rest, pain medication, physiotherapy and bracing, is considered the standard care in patients with symptomatic osteoporotic VCFs. Since the introduction of percutaneous vertebroplasty (PV), this minimal invasive therapy that involves injection of bone cement in the fractured vertebral body, has gained popularity to stabilize osteoporotic VCFs with resultant relief of associated local back pain. PV is considered an accepted pain therapy in many centres and acknowledged as a useful additional option in the care for patients with osteoporotic VCFs.

In 1984 PV was developed in France for the treatment of painful aggressive vertebral angioma [3]. In the following years the indications for PV were expanded to vertebral fractures caused by osteoporosis, trauma, malignant or benign vertebral tumors and vertebral osteonecrosis. Presently, PV is most frequently performed to treat patients with painful osteoporotic VCFs.

Many prospective and retrospective studies on PV have been published and described a high clinical success rate [4-12]. A recent systematic literature review demonstrated the effectiveness of PV in 87% of patients in terms of pain relief as well as a short- and long-term improvement of physical function [13]. However, these studies did not have a control group. In fact, the natural course of pain in similar patients with a VCF was unknown. Two non-randomised controlled trials have been published comparing PV with conservative therapy [14,15]. Both studies demonstrated a significant better improvement in pain scores after PV on the short-term. However, after 6 months no differences could be demonstrated. VERTOS, the first randomised controlled trial (RCT) with a limited group of 34 patients, reported only on short-term results [16]. Longer term results could not be obtained due to the cross-over of many patients from optimal pain medication to PV. VERTOS confirmed immediate pain relief and improvement of mobility, function and stature after PV. The short-term results after PV were significantly better compared to pain medication in these patients with sub-acute or chronic osteoporotic VCFs.

Recently, three RCTs concerning PV have been published with conflicting results. Investigators in two randomized controlled trials [17,18] concluded that there is no benefit to PV over a sham placebo procedure involving the injection of local anesthetic into the area adjacent to the fracture. In the study by Buchbinder et al [17], 78 patients with one or two painful osteoporotic VCFs were randomized to receive either PV or a sham procedure, which included infiltration of anesthetic into the pedicular periosteum. The primary measured outcome was overall pain at 3 months. Despite significant reductions in overall pain in both groups, there was no significant advantage of PV over the sham procedure. In the study by Kallmes et al [18], 131 patients with one to three painful osteoporotic VCFs were randomized to undergo either PV or a simulated sham procedure, which included infiltration of anesthetic into the periosteum of the posterior lamina. The primary outcomes were scores on the modified Roland-Morris Disability Questionnaire (RDQ) and average pain intensity during the preceding 24 hours at 1 month. Treatment group crossover was permitted at 1 month. At 1 month, there was no significant difference between the two groups in either the RDQ score or the pain rating. In the third RCT, VERTOS II [19], PV was compared to optimal conservative treatment in 202 patients who were 50 years or older, had vertebral compression fractures on spine radiograph, had experienced back pain for 6 weeks or less, and a visual analogue scale (VAS) score for pain of 5 or more. The primary outcome was pain relief at 1 month and 1 year as measured by VAS score. The authors concluded that in a subgroup of patients with acute osteoporotic vertebral compression fractures and persistent pain, percutaneous PV is effective and safe. Pain relief after PV is immediate, is sustained for at least a year, and is significantly greater than that achieved with conservative treatment, at an acceptable cost.

The most important differences between the two sham studies and VERTOS II is patient selection. In the sham studies both acute and chronic fractures were included while in VERTOS II only acute fractures were eligible. In addition, bone edema in the affected vertebra was not a consistent inclusion criterion in the sham studies. The sham studies lacked a control group without intervention. The discordant results from the sham studies, on the one hand, and VERTOS II, on the other hand, have incited much debate [20-24]. Apparently clinicians do still not know how to best treat their patients. The objectives of this study is to evaluate the efficacy of bone cement injection in PV for patients with acute painful osteoporotic compression fractures, as compared with a simulated placebo procedure without injection of bone cement. We hypothesize that patients who had undergone PV would report less pain at 1 day, 1 week and 1, 3, 6 and 12 months (the primary outcomes) than those in the sham control group. In addition, we hypothesize that the back pain related disability is less and the quality of life (QOL) is better of patients undergoing PV compared with patients in the simulated placebo condition

## Methods

### Study design

VERTOS IV is a multicenter RCT concerning the treatment of patients with a painful osteoporotic VCF. Patients are recruited on the Radiology departments of the participating hospitals and randomized to PV or a simulated procedure. Upon obtaining informed consent an independent central telephone operator completes the randomisation procedure, using a computer program. The maximum allowed unbalance (block size) is six, with a maximum sample size of 84 for each participating centre. A total of 180 patients will be enrolled, 90 in each group. This is based on the assumption of a 1.5 point difference in pain relief (VAS Score) and a 20% withdrawal rate (α = 0.05 and β = 0.20, 7 measurement points). The enrolment of patients will take place in four centres in The Netherlands: St. Elisabeth Ziekenhuis in Tilburg, Catharina Ziekenhuis in Eindhoven, Medisch Spectrum Twente in Enschede and Albert Schweitzer Ziekenhuis in Dordrecht. Randomization will start January 2011 with an expected completion of enrolment by January 2013. There is a one-year follow-up, with the possibility of an extended follow-up at two years.

The overall Institutional Review Board approval is obtained at the St. Elisabeth Hospital in Tilburg (NL33978.008.10). In addition, each participating centre will obtain a local Institutional Review Board Approval. This study is registered at ClinicalTrials.gov., NCT01200277.

### Patients

All patients, 50 years of age or older, referred for an X-ray of the thoracic and/or lumbar spine, receive a short clinical questionnaire. Patients diagnosed with a VCF (Th5-L5), pain for six weeks or less and a Visual Analogue Scale (VAS) score of five and higher are contacted to participate in the study. After informed consent patients undergo a magnetic resonance imaging (MRI) scan of the spine, bone densitometry, blood sample screening and a consultation of an internist. Patients enrolled in the study comply with the following inclusion criteria: (1) VCF on X-ray of the spine (minimal 15% loss of height) [25], (2) level of VCF Th5 or lower, (3) back pain ≤ 6 weeks at time of X-ray, (4) ≥ 50 years of age, (5) bone edema on MRI of the fractured vertebral body, (6) focal tenderness on VCF level and (7) decreased bone density T-scores ≤ -1. The exclusion criteria are: (1) severe cardio-pulmonary condition, (2) untreatable coagulopathy, (3) systemic or local infection of the spine (osteomyelitis, spondylodiscitis), (4) suspected alternative underlying disease (malignancy) (5) radicular or caudal compression syndrome and (6) contra-indication for MRI. All patients contacted to participate in the study are registered in order to obtain an overview of the total patient population.

### MRI protocol

MRI is performed prior to randomization using a 1, 1.5 or 3 Tesla MRI scanner. The following MRI sequences are employed: sagittal T1 (TR 400 ms, TE 13 ms), T2 Turbo Spin Echo (TR 3500 ms, TE 120 ms) and STIR (TR 2500 ms, TE 70 ms) and transverse T2 TSE (TR 2500 ms, TE 120 ms) at the level of the affected VCF. Bone edema in the VCF is defined as increased signal intensity at the STIR images and decreased signal intensity at the T1 weighted images. The shape and grade of every VCF is scored by two radiologists using the visual semiquantitative system according to Genant (26). When there is disagreement between both observers a consensus meeting is held. The shape of the VCF is classified as wedge, biconcave or crush, depending on whether anterior, middle or posterior portion of vertebral body is most diminished in height. The grade of VCF is classified as a percentage of height reduction in mild (15-25%), moderate (25-40%) and severe (>40%).

### Interventions

The pre-procedural work-up consists of: ECG, chest X-ray and blood sampling. One hour prior to the procedure 2 g cefazolin is administered intravenously as prophylaxis. Patients are brought to the angio suite, where sterile preparation for surgery is performed. Using fluoroscopic guidance, the practitioner infiltrates the skin and subcutaneous tissues overlying the pedicle of the target vertebra or vertebrae with 1% lidocaine and infiltrates the periosteum of the pedicles with 0.25% bupivacaine (marcaine). Patients are then randomly assigned to undergo either the full PV procedure or the sham intervention. For the PV procedure, 11-gauge or 13-gauge needles are passed into the central aspect of the target vertebra or vertebrae. Bone cement is prepared on the bench and injected under constant fluoroscopy into the vertebral body. Injection is stopped when the cement reaches to the posterior aspect of the vertebral body or leaks into an extraosseous space, such as the intervertebral disk or an epidural or paravertebral vein. During the sham intervention, verbal and physical cues, such as pressure on the patient's back, are given, and the methacrylate monomer is opened to simulate the odor associated with mixing of bone cement, but the needle is not placed and cement is not injected. After the procedure, all patients are monitored in the supine position for 1 to 2 hours before discharge. Cross-over will not be offered before 6 months after randomization.

### Osteoporosis- and pain medication

All patients receive osteoporosis medication, such as bisphosphonates together with supplemental calcium and vitamin D. Pain medication demanded by the patient is recorded. Analgesics are classified following the WHO classification: (1) Paracetamol (acetaminophen), (2) Tramadol, (3) Tramadol and Paracetamol, (4) Morphine. Non Steroid Anti Inflammatory Drugs (NSAID) are only prescribed if patients are intolerant for opiate-derivatives or when already used. Corrections in dose and classification of pain medication are made if necessary.

### Clinical follow-up

An experienced nurse-practitioner requests patients to complete a standard questionnaire before and at 1 day, 1 week, and 1, 3, 6 and 12 months after the procedure. The questionnaire consist of the VAS score and questions about use of pain medication, pain location, and pain type (primary outcomes). The VAS score is a pain score ranging from 0 (no pain) to 10 (worst pain ever) [26] Questions concerning use of pain medication, pain location, and pain type are included. Secondary outcomes are back pain related disability and QOL as measured with the Roland Morris Disability (RMD) Questionnaire and the Questionnaire of the European Foundation for Osteoporosis (Qualeffo), respectively. The Qualeffo is developed specifically for patients with osteoporosis [27]. This questionnaire consists of 41 questions about: pain, physical function, social function, general health perception, and mental function. The Qualeffo score ranges from 0 (best quality of life) to 100 (worst quality of life). This questionnaire will be completed at five measurement moments (before and at 1, 3, 6, and 12 months after the procedure). The RMD questionnaire is a disability questionnaire that measures the functional status of patients with back pain [28,29]. The RMD will be completed at all measurement points. Patients visit the internist at 1, 3, 6 and 12 months follow-up. Patients receive a pain diary. Patients are asked to complete the VAS score and use of analgesics is recorded on a daily basis during the first month after randomization.

### Sample size considerations

Conform Kallmes et al. [18], we expect a 1.5 point difference in pain relief as measured by VAS score. Given that there are seven measurement points (α = 0.05 and β = 0.20) we need 142 patients in total). Assuming a withdrawal rate of 20%, a total of 180 patients will be enrolled, 90 in each group. We used G-Power to calculate the sample size.

### Statistical analysis

The data will be analysed according to the intention-to-treat principle. Standard statistical techniques will be used to describe characteristics of patients in both groups. We will compare baseline characteristics in the two treatment groups and if incomparability appears, we will in secondary analysis, adjust for differences. The primary outcome, significant pain relief, will be compared between groups using analysis of variance for repeated measures. If adjustment for possible baseline incomparability is needed, analysis of covariance will be done. Since we expect that the difference between the two groups will become evident from 3 months onwards, the analysis will also be performed only using scores before and 3, 6, and 12 months after the procedure.

## Conclusion

The VERTOS IV study is a RCT comparing PV with a sham intervention using strict inclusion criteria designed to assess pain relief in patients with an acute osteoporotic VCF with mid-term follow-up.

## Competing interests

The authors declare that they have no competing interests.

## Authors' contributions

All authors conceived of the study and participated in the design. They all read and approved the final manuscript.
